# Pulp Extractors

**Published:** 1866-09

**Authors:** 


### PULP EXTRACTORS.

We have received beautiful samples of these little instruments, one package from Dr. J. S. Latimer, of New York City, and another from S. S. White. The instruments in these two packages are so nearly alike in every apparent respect that one would think them made by the same hand. They are very delicate and more perfectly barbed than any we have seen. They catch readily and hold tenaciously any thing in the shape of tooth pulp that may be brought in contact with them. They constitute a beautiful example of the progress that has been made, in comparatively a short time, in the manufacture of fine, delicate instruments for dental purposes.

The question is often asked, "Why do we not have finer instruments than the mass of those that are in the market?" The answer is simply, the profession does not demand them. It is true that a few fine operators obtain the best instruments possible, regardless of cost. These are exceptions. The mass of dentists buy cheap instruments—perhaps for a cheap dentist that is the best course: but for him who desires to go forward, it is *not* the best method. The more an instrument costs the better, so it is correspondingly good. We would say to our professional brethren, refuse to purchase of the manufacturers or dealers any thing but the most perfect instruments possible. Simply let the second and third-rate instruments lie upon the shelves, and demand that better be produced, and it will be done. We have no hesitation in saying that all dental instruments could be made far better than they are; then let us have it so.

We shall speak of this again.

